# Employment of Young Adult Cancer Caregivers, Other Disease Caregivers, and Non-Caregiving Adults

**DOI:** 10.3390/ijerph18147452

**Published:** 2021-07-13

**Authors:** Echo L. Warner, Andrew R. Wilson, Jessica G. Rainbow, Lee Ellington, Anne C. Kirchhoff

**Affiliations:** 1 University of Arizona Cancer Center, 1515 N Campbell Ave, Tucson, AZ 85724, USA; 2 University of Arizona College of Nursing, 1305 N Martin Ave, Tucson, AZ 85721, USA; jrainbow@arizona.edu; 3 Parexel International, 2520 Meridian Pkwy # 200, Durham, NC 27713, USA; Andrew.Wilson@nurs.utah.edu; 4 University of Utah College of Nursing, 10 2000 E, Salt Lake City, UT 84112, USA; lee.ellington@nurs.utah.edu; 5 Huntsman Cancer Institute, Cancer Control and Population Sciences, 2000 Cir of Hope Dr, Salt Lake City, UT 84112, USA; anne.kirchhoff@hci.utah.edu; 6 Department of Pediatrics, University of Utah, 295 Chipeta Way, Salt Lake City, UT 84108, USA

**Keywords:** adolescent and young adult, cancer caregiver, employment, caregiver, caregiving intensity

## Abstract

Young adults are increasingly taking on caregiving roles in the United States, and cancer caregivers often experience a greater burden than other caregivers. An unexpected caregiving role may disrupt caregiver employment, leading to lost earning potential and workforce re-entry challenges. We examined caregiving employment among young adult caregivers (i.e., family or friends) using the 2015 Behavioral Risk Factor Surveillance System (BRFSS), which included caregiving, employment, and sociodemographic variables. Respondents’ ages varied between 18 and 39, and they were categorized as non-caregivers (*n* = 16,009), other caregivers (*n* = 3512), and cancer caregivers (*n* = 325). Current employment was compared using Poisson regressions to estimate adjusted incidence rate ratios (aIRR) and 95% confidence intervals (95% CI), including gender-stratified models. We estimated employment by cancer caregiving intensity (low, moderate, high). Cancer caregivers at all other income levels were more likely to be employed than those earning below USD 20,000 (aIRR ranged: 1.88–2.10, all *p* < 0.015). Female cancer caregivers who were 25–29 (aIRR = 0.71, 95% CI = 0.51–1.00) and single (aIRR = 0.70, 95% CI = 0.52–0.95) were less likely to be employed than their counterparts. College-educated males were 19% less likely to be employed than high school-educated caregivers (95% CI = 0.68–0.98). Evaluating caregiver employment goals and personal financial situations may help identify those at risk for employment detriments, especially among females, those with lower educational attainment, and those earning below USD 20,000 annually.

## 1. Introduction

In the United States, there are 1.46 million cancer caregivers aged 18–34 [1]. Cancer caregiving is associated with greater caregiver burden (e.g., number caregiving hours, activities of daily living, instrumental activities of daily living) than caring for patients with other diseases [1]. Among other factors, caregiving burden influences cancer caregivers’ employment [2,3,4], and a quarter to a third take extended leave [3,4].

The number of working-age young adults engaged in caregiving continues to grow [1]. Caregiving among young adults is often unexpected, occurring simultaneously with other caregiving roles (e.g., for children, parents, grandparents) [5,6]. Engaging in high-intensity caregiving when young adults have yet to establish career stability may be detrimental to employment. We hypothesized that higher intensity caregiving and female gender would be negatively associated with employment among young adult caregivers, particularly for young adult cancer caregivers given their high levels of caregiver burden.

## 2. Materials and Methods

We used the 2015 Behavioral Risk Factor Surveillance System (BRFSS), a nationally representative computer-assisted survey of non-institutionalized adults in the United States that collects data on preventive health behaviors and risk factors [7]. The 2015 BRFSS is a publicly available combined telephone and landline survey, with a median response rate of 47.2%. The BRFSS and caregiver module are described elsewhere [8].

### 2.1. Participants and Outcome

There were 441,456 participants in the 2015 BRFSS; 24 states incorporated a 9-item caregiving module (*n* = 108,995). Of these, *n* = 20,187 were young adults aged 18–39. Caregivers were identified by the question “During the past 30 days, did you provide regular care or assistance to a family member who has a health problem or disability?” Cancer caregivers were identified by asking “What is the main health problem, long-term illness, or disability that the person you care for has?” We excluded respondents who were missing/refused for these questions. Our analytic sample included three mutually exclusive participant categories: *n* = 16,009 non-caregivers, *n* = 3512 other caregivers, and *n* = 325 cancer caregivers.

Our outcome was a binary variable indicating current employment for wages/self-employed versus unemployed/unable to work (including respondents who were out of work for one year or more, out of work for less than one year, and unable to work). Homemakers, students, and/or retirees were excluded from employment analyses (*n* = 3689).

### 2.2. Other Measures

Sociodemographic variables included age, gender, race/ethnicity, marital status, and education. The caregiving intensity composite assigns points for managing personal care (e.g., giving medications, feeding, dressing, bathing), household care (e.g., cleaning, finances, meals), and hours of care provided per week [1,9]. Points are summed (0–8 points) based on personal care (+3 points), household care (+3), personal care and household care (+4), and the number of hours per week engaged in caregiving (+1–4 assigned to the following categories 0–8, 9–19, 20–39, and ≥40 h) [9]. We categorized intensity as low (1–4 points), moderate (5 points), and high (6–8 points) [9].

### 2.3. Statistical Analysis

Complex survey weighting procedures were incorporated into all analyses. Descriptive statistics were calculated using raw counts and BRFSS weight-adjusted proportions. Sociodemographic factors were compared between cancer caregivers and non-caregivers using Pearson χ^2^ tests, and then between caregivers of other conditions and cancer caregivers. We estimated crude incidence rate ratios (IRR) and adjusted incidence rate ratios (aIRR) and 95% confidence intervals (95% CI) using survey-weighted Poisson regression models with robust standard errors for common outcomes [10]. We compared the employment of non-caregivers to other caregivers and cancer caregivers. Among cancer caregivers, we estimated models of employment by caregiving intensity, age, gender, race/ethnicity, marital status, education, and annual household income, and ran adjusted models stratified by gender. All data were analyzed using Stata 16.

## 3. Results

Cancer caregivers were less likely to be unemployed/unable to work than other caregivers (14.1% vs. 20.3%, Table 1). More cancer caregivers were older (18–24 years: 32.0% vs. 29.7%, 25–29 years: 22.2% vs. 19.2%, *p* < 0.001) than Other caregivers, but younger than non-caregivers (*p* < 0.001). Cancer caregivers tended to have lower incomes than non-caregivers (*p* = 0.036). Compared to cancer caregivers, other caregivers differed by age, education, and income (all *p* < 0.01).

Cancer caregivers’ employment did not differ significantly from non-caregivers or other caregivers; however, other caregivers were less likely to be employed than non-caregivers (IRR = 0.91, 95% CI = 0.89–0.94, *p* < 0.001), even after adjusting for age, gender, race/ethnicity, marital status, education, and annual household income (aIRR = 0.94%, CI = 0.91–0.97, *p* < 0.001, data not shown).

Among cancer caregivers, only income influenced the overall likelihood of employment, with caregivers from all income groups having higher likelihood of employment compared to those earning less than USD 20,000 annually (aIRR ranged: 1.88–2.10, all *p* ≤ 0.015, Table 2). However, female caregivers aged 25–29 were less likely than those aged 18–24 years to be employed (aIRR: 0.71, 95% CI = 0.51–1.00). Single female cancer caregivers were less likely to be employed than married/partnered female cancer caregivers (aIRR = 0.70, 95% CI = 0.52–0.95), and female cancer caregivers in all but the highest income bracket were more likely to be employed than those earning below USD 20,000 annually (all *p* ≤ 0.047). Among males, those with the highest educational attainment were significantly less likely to be employed (aIRR: 0.81, 95% CI 0.68–0.98) than the least educated. All models adjusted for age, race/ethnicity, marital status, education, and annual household income, and the overall model was also adjusted for gender.

## 4. Discussion

In this nationally representative sample, young adult caregivers were less likely to be employed than non-caregivers. Female young adult cancer caregivers who were single, aged 25–29 years, and those in households earning below USD 20,000 had lower likelihood of employment, as did males with the highest educational attainment. Sociocultural expectations for females to become caregivers may, in part, explain these differences. Caregiving that interferes with employment inflicts long-term detriments on caregivers’ careers and emotional wellbeing [4], and these effects may be pronounced for young adults and female cancer caregivers. As young adults increasingly engage in informal caregiving, supporting caregiver employment, a major component of economic stability, is a public health priority [11].

At a young age, women who leave the workforce to provide care may experience difficulty returning to work (e.g., lost or outdated skills, scheduling conflicts, overdue licensure), potentially impacting their financial stability. In our sample, single women, women from low-earning households, and those aged 25–29 were especially likely to be unemployed. Female cancer caregivers earning between USD 20,000 to USD <75,000 were more likely to be employed than the lowest earning caregivers, underscoring the need for flexible employment options that are not tied to educational attainment for these caregivers. Male cancer caregivers with the highest educational attainment were significantly less likely to be employed compared to those with high school education. High caregiving intensity had a significantly negative effect on employment among caregivers, but this was attenuated after adjusting for income (data not shown), potentially suggesting that higher earning caregivers have access to resources that mitigate the influence of high-intensity caregiving on employment that lower earning caregivers do not. Cumulative employment impacts resulting from lack of flexible work schedules, family leave, and paid time off for caregiving may restrict young cancer caregivers’ workforce retainment [12,13].

The BRFSS does not consider preferences for full versus part-time work, the toll of working while caregiving (i.e., presenteeism), nor caregivers’ desires for workforce participation. Hispanic and African American caregivers disproportionately report financial and employment burdens;^2^ this sample may underrepresent their employment impacts, but this is a critical area for future study.

## 5. Conclusions

Federal policies and certain state policies provide limited employment accommodations for young caregivers. More robust support is needed to mitigate the negative effect of caregiving on young adults’ employment, especially for single females and those from low-earning households. Policies that support high-quality employer flexibility and educational attainment may protect young adult caregivers from negative employment changes, especially for female young adult cancer caregivers.

## Figures and Tables

**Table 1:** Sociodemographic factors for young adult cancer caregivers, non-caregivers, and other caregivers.

	Cancer Caregivers *n* = 325		Non-Caregivers *n* = 16,009			Other Caregivers *n* = 3512		
	N	% ^1^	N	% ^1^	*p*-Value ^2^	N	% ^1^	*p*-Value ^3^
**Employment status ^6^**								
Employed for wages/self-employed	230	85.9	11,462	85.9	0.990	2517	79.7	**<0.001**
Unemployed/unable to work	43	14.1	1659	14.1		572	20.3	
Out of work for 1 year or more ^5^	12	43.2	430	29.3	0.222	168	29.4	0.562
Out of work for less than 1 year ^5^	14	23.6	619	40.2		225	42.5	
Unable to work ^5^	17	33.2	610	30.5		179	28.1	
**Age**								
18–24	74	32.0	4618	34.0	**<0.001**	684	29.7	**<0.001**
25–29	72	22.2	3475	20.2		620	19.2	
30–34	86	26.3	4082	24.1		828	26.4	
35–39	93	19.4	4650	21.7		957	24.7	
**Gender**								
Female	126	56.4	7687	49.4	0.138	1750	48.6	0.463
Male	199	43.6	9138	50.6		1339	51.4	
**Race/ethnicity**								
Non-Hispanic White	212	67.1	11,220	65.0	0.642	1950	64.4	0.583
Other	110	32.9	5412	35.0		1092	35.6	
**Marital status**								
Married/Partnered	149	41.7	8024	43.0	0.766	1357	41.6	0.263
Unmarried	176	58.3	8733	57.0		1724	58.4	
**Education ^4^**								
≤High school graduate	119	46.0	5766	42.2	0.675	1139	46.7	**<0.001**
Some college/technical	102	31.2	5073	33.4		1080	34.5	
≥College graduate	104	22.9	5958	24.4		866	18.8	
**Annual household income (USD)**								
Less than $20,000	57	15.6	2472	18.2	**0.036**	583	22.1	**<0.001**
$20,000 to $34,999	66	22.8	2848	20.6		644	25.0	
$35,000 to $49,999	53	23.4	2196	15.1		384	14.2	
$50,000 to $74,999	29	8.5	2337	15.4		397	14.9	
$75,000 or more	75	29.7	4423	30.7		635	23.7	
**Caregiving intensity**								
Managing personal care								
Yes	196	57.8	-	-		1689	53.6	0.149
No	128	42.2	-	-		1357	46.4	
Managing household care								
Yes	274	84.5	-	-		2495	81.9	0.238
No	50	15.5	-	-		553	18.1	
Hours of care provided per week								
Up to 8 h	188	57.8	-	-		1810	60.1	0.782
9–19 h	49	15.1	-	-		417	14.6	
20–39 h	41	10.1	-	-		295	10.2	
40 h or more	35	17.0	-	-		445	15.1	
**Caregiving Intensity Composite**								
Low	108	40.8	-	-		1184	45.8	0.479
Moderate	83	30.2	-	-		681	24.4	
High	86	29.0	-	-		772	29.8	

^1^ Weighted for BRFSS sampling. ^2^ Weighted chi-square test of independence comparing cancer caregivers to non-caregivers, bold indicates significance at *p* < 0.05. ^3^ Weighted chi-square test of independence comparing cancer caregivers to other caregivers, bold indicates significance at *p* < 0.05. ^4^ Education missing for *n* = 28 non-caregivers and *n* = 4 non-cancer caregivers. ^5^ Weighted proportions represent only those who were unemployed. ^6^ Totals do not equal column headers because homemakers, students, and/or retirees were excluded from employment analyses (*n* = 3689).

**Table 2:** Factors associated with employment among all young adult cancer caregivers and by gender.

	Full Sample ^1^			Gender-Stratified Models							
				Female ^2^				Male ^2^			
	aIRR	95% CI	*p*-Value	%	aIRR	95% CI	*p*-Value	%	aIRR	95% CI	*p*-Value
Caregiving intensity ^1^											
Low	Ref.			37.1	Ref			45.3	Ref		
Moderate	0.95	0.86–1.06	0.374	26.5	0.88	0.71–1.08	0.218	34.7	0.98	0.87–1.11	0.796
High	0.84	0.70–1.00	0.056	36.3	0.74	0.55–1.00	0.054	20.1	0.97	0.83–1.14	0.734
Age											
18–24	Ref.			26.2	Ref.			39.8	Ref.		
25–29	0.89	0.69–1.16	0.408	24.3	**0.71**	**0.51–1.00**	**0.049**	19.3	1.21	0.98–1.50	0.069
30–34	1.12	0.97–1.29	0.107	28.5	1.21	0.92–1.59	0.182	23.5	1.11	0.92–1.35	0.265
35–39	0.99	0.85–1.14	0.854	21.0	1.04	0.77–1.42	0.775	17.4	1.07	0.86–1.34	0.531
Gender											
Male	Ref.										
Female	0.99	0.89–1.11	0.834		-	-	-		-	-	-
Race											
Non-Hispanic white	Ref.			67.8	Ref.			68.8	Ref.		
Other	1.02	0.89–1.18	0.729	32.2	0.97	0.75–1.24	0.797	31.2	1.12	0.97–1.30	0.112
Marital status											
Married/partnered	Ref.			49.6	Ref.			35.7	Ref.		
Single	0.89	0.78–1.02	0.101	50.4	**0.70**	**0.52–0.95**	**0.020**	64.3	0.97	0.86–1.10	0.681
Education											
≤High school	Ref.			38.7	Ref.			55.5	Ref.		
Some college	0.92	0.78–1.09	0.334	31.5	0.99	0.72–1.37	0.962	30.7	0.92	0.80–1.06	0.235
≥College graduate	1.03	0.86–1.23	0.74	29.8	1.18	0.92–1.53	0.195	13.8	**0.81**	**0.68–0.98**	**0.028**
Annual household income (USD)											
Less than $20,000	Ref.			24.0	**Ref.**			5.3	Ref.		
$20,000 to $34,999	**1.95**	**1.20–3.18**	**0.007**	22.0	**1.88**	**1.15–3.06**	**0.012**	24.2	1.88	0.68–5.19	0.222
$35,000 to $49,999	**1.88**	**1.14–3.11**	**0.014**	12.7	**1.66**	**1.01–2.74**	**0.047**	36.3	2.09	0.75–5.81	0.157
$50,000 to $74,999	**2.10**	**1.28–3.47**	**0.004**	9.2	**2.01**	**1.19–3.41**	**0.009**	7.6	1.95	0.68–5.53	0.211
$75,000 or more	**1.92**	**1.13–3.27**	**0.015**	32.1	1.69	0.99–2.88	0.054	26.7	2.16	0.79–5.89	0.133

^1^ Adjusted for age, gender, race/ethnicity, marital status, education, and annual household income. BRFSS weights applied. Bold indicates significance at *p* < 0.05. ^2^ Adjusted for age, race/ethnicity, marital status, education, and household income. BRFSS weights applied. Intensity missing/refused for *n* = 3.

## Data Availability

The BRFSS dataset is publicly available at BRFSS Survey Data and Documentation. Available online: https://www.cdc.gov/brfss/annual_data/annual_2015.html (accessed on 7 July 2021).
